# Preclinical Pharmacokinetic Evaluation of Mithramycin and Mithramycin SA Tryptophan-Conjugated Analog

**DOI:** 10.3390/pharmaceutics17060765

**Published:** 2025-06-10

**Authors:** Kumar Kulldeep Niloy, Jamie Horn, Nazmul H. Bhuiyan, Khaled A. Shaaban, Suhas S. Bhosale, Thomas E. Prisinzano, Jon S. Thorson, Jurgen Rohr, Markos Leggas

**Affiliations:** 1 Department of Pharmacy and Pharmaceutical Sciences, St. Jude Children’s Research Hospital, Memphis, TN 38105, USAjamie.horn@stjude.org (J.H.); 2 Department of Pharmaceutical Sciences, College of Pharmacy, The University of Tennessee Health Science Center, Memphis, TN 38163, USA; 3 Department of Pharmaceutical Sciences, College of Pharmacy, University of Kentucky, Lexington, KY 40536, USA; nbhuiyan@adesisinc.com (N.H.B.); khaled_shaaban@uky.edu (K.A.S.); suhas.bhosale@uky.edu (S.S.B.); prisinzano@uky.edu (T.E.P.); jsthorson@uky.edu (J.S.T.); jurgen.rohr@uky.edu (J.R.); 4 Center for Pharmaceutical Research and Innovation, College of Pharmacy, University of Kentucky, Lexington, KY 40536, USA

**Keywords:** mithramycin, mithramycin analogs, preclinical pharmacokinetics, plasma protein binding

## Abstract

**Background**: Mithramycin (MTM) is a polyketide anti-cancer natural product previously identified as an EWS-FLI1 inhibitor. This oncogenic transcription factor is a canonical target for drug development in Ewing sarcoma. However, poor pharmacokinetics have been identified as a critical liability of MTM, preventing its further development. Through semisynthetic chemical modifications, we identified mithramycin SA-Trp (MTMSA-Trp) as being a pharmacologically superior congener. To explore their pharmacokinetic (PK) differences, this study examined the plasma PKs and plasma protein binding (PPB) of MTM and MTMSA-Trp in mice, rats, and monkeys. **Methods**: Protein binding was investigated by rapid equilibrium dialysis in plasma from mice, rats, monkeys, and humans. The pharmacokinetics were investigated at milligram- and microgram-level doses in mice and rats. The pharmacokinetics in monkeys were investigated using the cassette dosing approach at two microgram-level doses. The MTMSA-Trp pharmacokinetic linearity was evaluated in mice at 0.3, 1, 3, and 10 mg/kg doses. All samples were analyzed using LC-MS/MS. **Results**: Plasma protein binding was higher for MTMSA-Trp (1–4% unbound) than for MTM (10–30% unbound) across species, except in athymic nude mice (1–4% unbound and <1% for mithramycin and MTMSA-Trp, respectively). In mice and rats, MTMSA-Trp had significantly lower clearance than MTM at both milligram and microgram doses; however, the difference in plasma exposure was more pronounced at milligram doses. Consistent with the rodent PK results, cassette microdosing in monkeys showed that the clearance of MTMSA-Trp was lower than that of MTM, but the differences were less pronounced. In the dose proportionality study, MTMSA-Trp showed linear pharmacokinetics at 1, 3, and 10 mg/kg doses. **Conclusions**: MTMSA-Trp has significantly lower clearance than MTM in rodent models. This is a significant improvement compared to the parent drug, MTM, and warrants further evaluation of PKs in non-rodent models to enable the prediction of MTMSA-Trp PK in humans.

## 1. Introduction

Ewing sarcoma is a malignant bone or soft tissue cancer that primarily occurs in children, adolescents, and young adults [1,2]. It is characterized by chromosomal translocations resulting in the expression of EWS-ETS fusion proteins, which function as oncogenic transcription factors [1,2]. Two fusion products, EWS-FLI1 and EWS-ERG, have been identified in approximately 85–90% and 10–15% of Ewing sarcoma cases, respectively [1,2]. Due to their high occurrence and capacity to drive malignant transformation and progression, inhibiting EWS-ETS transcription factors is regarded as a viable target for drug development campaigns in Ewing sarcoma [3,4,5,6,7].

MTM was identified as a potent inhibitor of EWS-FLI1 through high-throughput screening conducted at the National Cancer Institute [6]. However, the poor PKs and dose-limiting toxicities observed in a subsequent pediatric phase I/II clinical trial have prevented its further clinical use [7,8]. This finding led us to consider diverse structural modifications of MTM achieved via semisynthetic approaches to identify analogs with improved therapeutic windows and PK profiles compared to MTM. In this context, modifications in the C3-side chain of MTM were found to be an effective strategy, leading to two classes of MTM analogs: MTMSA and MTM oximes [4,5]. These analogs have increased lipophilicity and are expected to have higher plasma protein binding, which may lead to prolonged plasma exposure compared to that of MTM [5]. Unlike MTM, MTMSA has a shorter side chain terminating in a carboxylic acid [9], which allows for the semisynthetic generation of analogs.

Cell-based and molecular assays identified the tryptophan-conjugated analog of MTMSA, MTMSA-Trp, as having improved pharmacological properties, including superior potency and target engagement and lower off-target toxicity than MTM [4]. This led to ongoing efforts to evaluate MTMSA-Trp as a lead candidate for preclinical development [10,11]. In this regard, PK assessment in preclinical species is a crucial step towards the further development of MTMSA-Trp, since poor PKs have been identified as a critical liability of MTM. Considering the increased lipophilicity resulting from structural modifications of this class of compounds, it is essential to evaluate whether the enhanced lipophilicity of MTMSA-Trp leads to higher protein binding and, consequently, a more favorable PK profile compared to that of MTM.

To understand the PK differences across preclinical species, this study aimed to evaluate and compare the PKs of MTM and MTMSA-Trp in mice, rats, and monkeys. Additionally, plasma protein binding studies were performed using plasma from these species and humans to understand the potential impact of increased lipophilicity on plasma protein binding and PKs. The preclinical PK characterization of MTMSA-Trp will facilitate future efforts to construct physiologically based PK models that may predict the clearance of the compound in humans. This has implications for future trial designs but may also provide critical information while considering the advancement of this compound toward the clinic.

## 2. Materials and Methods

### 2.1. Test Compounds and Animals

Mithramycin, from *Streptomyces argillaceus*, of 98.5% purity or higher, was purchased from Fermentek, Ltd. (Jerusalem, Israel). Following a previously published semisynthetic scheme, its tryptophan-conjugated analog, MTMSA-Trp, was synthesized and purified to ≥ 97.1% at the University of Kentucky Center for Pharmaceutical Research and Innovation [4,12]. Unless otherwise indicated, the test compounds were formulated with excipients and solvents purchased at the highest grades available from Sigma-Aldrich (St. Louis, MO, USA) in buffers created using the highest salt grades available from Fisher Scientific (Fair Lane, NJ, USA) and syringe-filtered for sterilization (0.2 µm pore-sized, 25 mm diameter nylon syringe filters from Fisher Scientific, Fair Lane, NJ, USA). The analytical solvents and additives were Optima^TM^ LC/MS grade, acquired from Fisher Scientific (Fair Lane, NJ, USA).

Mouse and rat experiments were performed at St. Jude Children’s Research Hospital under an institutionally approved protocol (St. Jude Institutional Animal Care and Use Committee protocol 3164, approved 29 March 2022). Additionally, mouse and rat experiments were performed at the University of Kentucky under institutionally approved protocols (University of Kentucky Institutional Animal Care and Use Committee protocols 2016-2345, approved 14 February 2019, and 2018-2849, approved 15 June 2021). Non-human primate experiments were performed at the University of Kentucky under institutionally approved protocols (University of Kentucky Institutional Animal Care and Use Committee protocols 2018-3033, approved 15 August 2018). Athymic nu/nu mice and Sprague Dawley rats were purchased from Envigo RMS (Indianapolis, IN, USA). Adult male Cynomolgus macaques of Vietnamese origin were obtained from Covance (Alice, TX, USA). The animals were housed in AAALAC-certified, temperature- and humidity-controlled (64° F to 84° F and 30% to 70%, respectively) facilities with light/dark cycles of 12 h each. All animals were provided with standard commercial diets (rodent 5R53 TestDiet, Richmond, IN, USA, or primate Teklad Global^TM^ 20% Protein, Inotiv, Indianapolis, IN, USA) and chlorinated, reverse-osmosis-treated water was available ad libitum. The primate diets were supplemented with fruits and vegetables.

### 2.2. Plasma Protein Binding

Protein binding was assessed in plasma derived from athymic nude (nu/nu) mice, Sprague Dawley rats, Cynomolgus monkeys, and humans. Plasma from athymic nude mice and Sprague Dawley rats was collected from animals housed locally. For other species, whole blood was purchased from BioIVT (Westbury, NY, USA). Plasma was collected by centrifuging the whole blood immediately upon collection, stored at 4 °C, and used within one week of storage for protein binding studies. The plasma was spiked with the compound to prepare 1, 3, and 10 µM final concentrations (0.1% DMSO (*v*/*v*)). Spiked plasma (100 µL) and PBS (350 µL) were placed in their respective chambers in the Pierce™ RED Device (12K MWCO; Linden Bioscience, Research Triangle Park, NC, USA) and incubated in a Talboys microplate shaker at 37 °C and 250 rpm for 4 h to achieve equilibrium. Post-dialysis samples were collected from the plasma and buffer chambers, matrix-matched with PBS and plasma, respectively, and analyzed by LC-MS/MS as detailed below. The experiments were performed in triplicate, and the unbound percentage was calculated based on the following equation [13]:

$$f_{u}=\frac{PBS\;chamber\;area\;ratio}{Plasma\;chamber\;area\;ratio};Area\;ratio=\frac{analyte\;area}{ISTD\;area}$$

### 2.3. Pharmacokinetic Studies

PK studies were conducted in nu/nu mice, Sprague Dawley rats, and Cynomolgus monkeys. Both male and female nu/nu mice were used for MTMSA-Trp, and male nu/nu mice were used for MTM. Male rats and monkeys were used for both MTM and MTMSA-Trp. All PK studies are listed and summarized in Table 1. In the initial studies, MTM and MTMSA-Trp were formulated in 2.5% Kolliphor EL/97.5% PBS (*v*/*v*). Subsequently, a formulation of 5% Solutol HS-15 (MedChemExpress, Monmouth Junction, NJ, USA)/95% PBS at pH 8 (*v*/*v*) was utilized to accommodate the higher dosing of either compound. This formulation was also used for microdose PK studies in mice and rats. The dosing solutions were typically used within 1–2 days and no later than a week following formulation. A stability assessment of MTM indicated that this drug remained stable for up to 550 days in the various formulations used in these experiments. MTMSA-Trp dosing solutions were stable for at least 11 days following formulation. All dosing was performed via intravenous (IV) bolus tail vein injection in mice and rats and by IV infusion (10 min) in monkeys. The varying numbers of animals and blood collection methods employed across the different PK studies in mice were based on the study objectives and ethical considerations aimed at minimizing animal usage. Serial sampling via saphenous vein bleeding was used in studies that evaluated the plasma PKs only. Saphenous bleeding did not require anesthesia. Destructive sampling was performed through cardiac puncture under isoflurane anesthesia. This approach was employed in studies where in addition to investigating the plasma PKs, tissue samples were collected for either toxicity or biodistribution analysis purposes, which are beyond the scope of this manuscript.

#### 2.3.1. Pharmacokinetic Studies in Mice

Four separate PK studies were conducted using nu/nu mice. For studies using destructive sampling, the animals were anesthetized under isoflurane gas and samples were collected through intracardiac puncture in heparinized syringes. The first mouse PK study (Study 1; Table 1) used destructive sampling. Male nu/nu mice (n = 15) were administered MTM or MTMSA-Trp at 2 mg/kg. Blood samples were collected (n = 3 mice/time point) by cardiac puncture at 5 and 30 min and at 1.5, 3, and 6 h. The second, third, and fourth mouse PK studies (Studies 7, 8, and 9; Table 1) were performed to assess the dose proportionality. The second mouse PK study (Study 7, Table 1) used destructive sampling. Female nu/nu (n = 12) mice were administered MTMSA-Trp at 1 mg/kg, 3 mg/kg, and 10 mg/kg. Blood samples (n = 3 mice/time point) were collected by cardiac puncture at 5 min, 30 min, 2 h, and 5 h. The third mouse PK study (Study 8; Table 1) sampled mice twice via the saphenous vein, with a final destructive sample through cardiac puncture. Three groups (n = 9/group) of nu/nu mice were dosed at 0.3 mg/kg, 1 mg/kg, and 3 mg/kg. The mice at each dose level were randomized into three groups of three mice. The sampling time points for the three doses were as follows: 10 min, 20 min, 30 min, 45 min, 1 h, 1.5 h, 2 h, 2.5 h, and 3 h for the 0.3 mg/kg group; 5 min, 15 min, 30 min, 1 h, 2 h, 3 h, 4 h, 6 h, and 8 h for the 1 mg/kg group; and 5 min, 15 min, 30 min, 1 h, 2 h, 4 h, 6 h, 9 h, and 12 h for the 3 mg/kg group. The fourth mouse PK study (Study 9, Table 1) used destructive sampling. Female nu/nu (n = 10) mice were administered MTMSA-Trp at 5 mg/kg. Blood samples (n = 2 mice/time point) were collected by cardiac puncture at 10 min, 30 min, 1 h, 3 h, and 6 h. After collection, blood samples were immediately centrifuged at 4000 g for 2 min, and plasma samples were immediately collected and placed on dry ice, followed by storage at −80 °C until analysis.

#### 2.3.2. Pharmacokinetic Studies in Rats

Rat PK studies were conducted using male Sprague Dawley rats (Study 2; Table 1). The rats were administered MTM (n = 6) or MTMSA-Trp (n = 6) at 0.5 mg/kg. Blood samples were collected at three different time points by saphenous bleeding. The collection time points were at 5 min and 20 min and at 1, 3, 6, and 9 h. The blood samples were processed and stored as described above.

#### 2.3.3. Pharmacokinetic Studies in Monkeys

Two separate cassette dosing PK studies were conducted using male cynomolgus monkeys. In the first monkey PK study (Study 3; Table 1), the primate was administered MTM and MTMSA-Trp at 6.94 µg/kg (n = 1) and 1.34 µg/kg (n = 1), respectively, in a cassette by a 10 min IV infusion. Blood samples were collected by peripheral venipuncture at 5 min, 15 min, 30 min, 1 h, 2 h, 3 h, 4 h, 6 h, and 8 h for MTM and at 5 min, 15 min, 30 min, and 1 h for MTMSA-Trp. In the second monkey PK study (Study 4; Table 1), the animal was administered MTM and MTMSA-Trp at 13.18 µg/kg (n = 1) and 4.19 µg/kg (n = 1), respectively, in a cassette by a 10 min IV infusion. Blood samples were collected by peripheral venipuncture at 5 min, 15 min, 30 min, 1 h, 2 h, 3 h, 4 h, 6 h, and 8 h for MTM and at 5 min, 15 min, 30 min, 1 h, and 2 h for MTMSA-Trp. The blood samples were processed and stored as described above.

#### 2.3.4. Allometric Scaling

Single-species allometric scaling was used to estimate the doses for mouse and rat microdose PK studies (Studies 5 and 6; Table 1). Mouse and rat doses equivalent to a 12 µg/kg monkey dose were calculated using the following allometric scaling equation [14].

$${Dose}_{animal}\;\left(mg/kg\right)={Dose}_{monkey}\;(mg/kg)\times {\left(\frac{{Weight}_{monkey}\;(kg)}{{Weight}_{animal}\;(kg)}\right)}^{0.33}$$

### 2.4. LC-MS/MS Analysis

All samples were processed and analyzed by LC-MS/MS using a bioanalytical method validated according to the FDA’s Bioanalytical Guidance, essentially as described by Eckenrode et al. with some modifications [15]. Briefly, the calibration curves ranged from 1 to 10,000 ng/mL for MTM and 5 to 10,000 ng/mL for MTMSA-Trp [15]. Plasma samples above the calibration curve range were diluted, along with quality control (QC) samples, and values were accepted when the interpolated concentrations were within 15% of the nominal values. The expected % recovery for both compounds was >90% [15,16]. Analyte separation and detection were performed on a Sciex Exion HPLC system equipped with a C18 analytical column (Phenomenex Synergi 4 µm Fusion-RP 80A; 50 mm × 2 mm with a guard column) and coupled to a hybrid triple-quadrupole/linear ion trap mass spectrometer (Sciex QTrap 6500+ or 7500+, Framingham, MA, USA). A 0.6 mL/min gradient of 0.005% formic acid in water and 0.005% formic acid in acetonitrile was used to elute the analytes within a 5 min time window. The effluent was volatilized under negative-mode electrospray ionization (4500 V spray voltage, 600 °C, 55 psi nebulizing N_2_, 70 psi heater N_2_, 40 psi curtain N_2_) and analyzed for the specific transitions of *m*/*z* 1083 → 935 (MTM; 1.7 min), *m*/*z* 1225 → 531 (MTMSA-Trp; 3 min), and *m*/*z* 1356.5 → 1006.5 (MTM_ox_25E, internal standard; 2.5 min). The internal standard was the MTM oxime–tryptophan [5]. The instruments were controlled and the data were integrated and processed using Sciex OS software version 3.3.1.43.

### 2.5. Pharmacokinetic Data Analysis

Noncompartmental analysis (NCA) was performed using PKanalix version 2024R1 (SimulationsPlus, Lancaster, CA, USA) to estimate the PK parameters from MTM and MTMSA-Trp concentration–time profiles. Only concentrations above the limit of quantification were used for NCA. The linear-up log-down trapezoidal integration method was used to estimate the area under the curve from time zero to the last measurable concentration–time point (AUC_last_). The terminal elimination rate constant (k_e_) was estimated by log-linear regression using the last three data points of the concentration–time profiles. Other PK parameters, such as the area under the curve from time zero extrapolated to infinity (AUC_0–inf_), the systemic clearance (CL), and the steady-state volume of distribution (V_d_), were estimated using the following equations [17].

$${AUC}_{inf}={AUC}_{last}+\frac{C_{last}}{k_{e}}$$

$$CL=\frac{Dose}{{AUC}_{inf}}$$

$$V_{d}={MRT}_{inf}\times CL$$

where C_last_ is the last measurable concentration–time point and MRT_inf_ is the mean residence time from time zero to infinity.

## 3. Results and Discussion

### 3.1. Plasma Protein Binding

To evaluate the putative effect of the increased lipophilicity of MTMSA-Trp due to the structural modification of MTM, in vitro plasma protein binding assays were performed on mouse, rat, monkey, and human plasma. The plasma protein binding results are shown in Table 2. The unbound fraction of MTMSA-Trp in all species was lower than that of MTM. The unbound fraction of MTM ranged from 10 to 20% across the concentrations (1, 3, and 10 µM) tested in the plasma from Sprague Dawley rats, Cynomolgus monkeys, and humans, except for nu/nu plasma, where the unbound fraction was approximately 1–5% at the tested concentrations. In this study, MTM plasma protein binding in human plasma was estimated to be ~81–83% (17–19% unbound), in the range of a previously reported value [16]. Conversely, the unbound fraction of MTMSA-Trp was between approximately 1 and 4% in Sprague Dawley rats and Cynomolgus monkeys. However, the unbound fraction in both nu/nu and human plasma was estimated to be <1%. The overall increase in the plasma protein binding of MTMSA-Trp can be partially attributed to the increased lipophilicity of this compound due to the hydrophobic nature of the conjugated tryptophan amino acid [18]. Such a positive relationship between lipophilicity and drug–protein binding is well-documented in the literature [19,20,21].

### 3.2. Pharmacokinetic Studies

**Figure 1:**
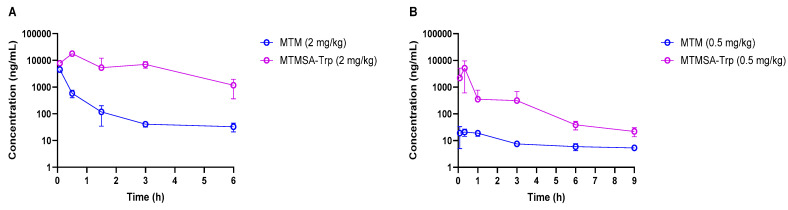
Concentration–time profiles of MTM and MTMSA-Trp in mice (**A**) and rats (**B**).

**Figure 2:**
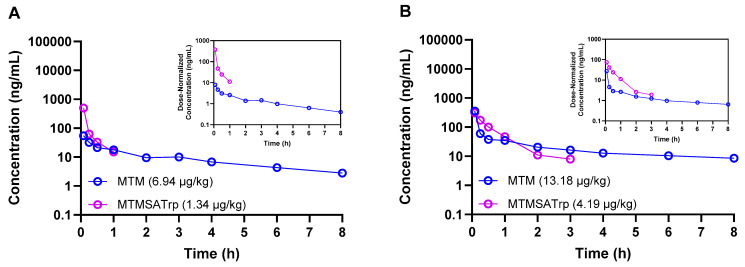
Concentration–time profiles of MTM and MTMSA-Trp co-administered (cassette dosing) to two male Cynomolgus monkeys at low (**A**) and high (**B**) microdose levels.

Studies were conducted in mice, rats, and monkeys at various doses to determine and compare the plasma PKs between MTM and MTMSA-Trp. The concentration–time profiles of both compounds are provided in Figure 1 and Figure 2, and the PK parameters are listed in Table 3 and Table 4.

PK experiments (Studies 1 and 2; Table 1) in male athymic nude mice and Sprague Dawley rats were conducted following IV bolus administration at 2 mg/kg and 0.5 mg/kg, respectively. Compared to MTM, the plasma exposure of MTMSA-Trp was considerably higher in both species (Figure 1A,B and Table 3). Significantly, the relative initial concentrations of MTM compared to those of MTMSA-Trp were much lower in rats than in mice, suggesting a higher volume of distribution. A possible explanation for this significant difference (Figure 1B) could be related to the clearance mechanism. Based on its structure, MTM and its analogs are predicted to be class 3B compounds, according to the extended clearance classification system, which suggests that they may be substrates of hepatic organic anion-transporting polypeptides (OATPs) [5]. This is plausible as it is known that MTM causes liver toxicity in humans, and animal studies suggest that toxicity is mediated through the disruption of bile acid homeostasis [7]. Having lower protein binding, MTM may partition more rapidly into the liver via uptake OATP transporters. In contrast, the highly bound MTMSA-Trp, although it may also be a substrate for uptake transporters, is restricted in the plasma compartment and partitions more slowly. While the initial concentration differences were not as pronounced in mice (Figure 1A), they followed a similar pattern. In this study, this was masked by technical variability. It was observed that the plasma concentrations in one or more samples collected at 5 min were considerably lower than those in 30 min samples, which decreased the average concentrations at 5 min in both species. This may have happened due to incomplete tail vein injection, where a part of the administered dose remains in the vein. However, this did not affect the overall PK profile of MTMSA-Trp and the overall conclusion of the study regarding the relative difference in the PKs between MTM and MTMSA-Trp. The PK parameters were estimated by NCA, and the systemic clearance of MTM was approximately 20-fold higher than that of MTMSA-Trp in male nu/nu mice (Table 3 and Figure 1A). Similarly, an approximately 20-fold higher systemic clearance was observed for MTM compared to MTMSA-Trp following the administration of a 0.5 mg/kg IV bolus dose in male Sprague Dawley rats (Table 3). In mice and rats, the lower systemic clearance of MTMSA-Trp led to higher plasma exposure, as estimated by comparing the AUC_0–inf_ (Table 3). The differences in MTM’s PK parameters between mice and rats (Table 3) may also be attributed to interspecies differences in substrate–orthologous uptake transporter interactions. Overall, the observation of the differences in the MTM PKs between rats and mice is potentially important and warrants further investigation with regard to toxicity modeling. It is likely that rats could be a better model to assess liver toxicity, with the expectation that they would more faithfully recapitulate the liver toxicity observed in humans and serve as a control to improve upon these effects with new analogs.

The terminal elimination half-lives ranged from ~1.5 to 12 h for both compounds, with MTM exhibiting a significantly longer terminal half-life in rats (Table 3). However, the longer half-life of MTM was not significant, as its terminal concentrations were very low compared to those of MTMSA-Trp. The terminal half-life (0.693/k_e_) was calculated based on log-linear regression using the last three concentration–time points. In the MTM rat PK study, the terminal concentrations were considerably low, leading to the regression line approaching a horizontal line, resulting in a smaller k_e_. Consequently, this produced a longer terminal half-life.

The inverse relationship between systemic clearance and plasma protein binding is known, where a decrease in plasma protein binding, in general, can lead to increased plasma clearance [22,23]. The faster clearance of MTM compared to MTMSA-Trp in mice and rats can, therefore, be explained partially by the lower plasma protein binding of MTM. In this regard, the low systemic clearance of MTMSA-Trp poses a significant advantage over other recently developed MTM analogs, such as MTM-SK, MTM-SDK, and EC-8042, which also demonstrated the same high systemic clearance as MTM [3,5,7,24,25]. The lower systemic clearance of MTMSA-Trp would allow for higher and more prolonged plasma exposure and offer less frequent dosing schedule options if indicated for mechanistic reasons. This is also supported by our previous finding, which demonstrated that the similar structural modification of MTM produced an analog, MTM_ox_32E, which had low systemic clearance, purported to be an effect of increased plasma protein binding due to increased lipophilicity [5]. However, the consequences of high plasma protein binding and the resulting low unbound fraction of MTMSA-Trp will need to be investigated further with prospective efficacy studies.

Having observed these differences in rodents, we next sought to estimate the PKs of both compounds in male Cynomolgus macaque monkeys, the rationale being that this species may provide useful data that can be incorporated into physiologically based pharmacokinetic models for human PK predictions. Given the difference in the in vitro cytotoxicity between MTM and MTMSA-Trp, as well as the known in vivo toxicity of MTM but unknown in vivo toxicity profile of MTMSA-Trp, we opted to administer the compounds at two escalating microdose levels using cassette dosing. MTM and MTMSA-Trp were first co-administered to one animal at 6.94 and 1.34 µg/kg, respectively. There was no observed toxicity, and a second animal was dosed with 13.18 and 4.19 µg/kg of MTM and MTMSA-Trp, respectively. These exploratory studies showed similar results to those of the rodent studies. A higher volume of distribution was estimated for MTM, and the clearance of MTMSA-Trp was lower, but the difference relative to the results for MTM was 2.5~7-fold rather than ~20-fold as in the case of rodents (Table 3).

**Figure 3:**
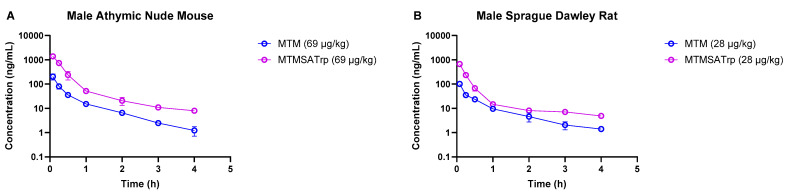
Comparison of MTM and MTMSA-Trp concentration–time profiles in (**A**) mouse and (**B**) rat at doses allometrically equivalent to 12 µg/kg in monkey.

As the degree of the difference in clearance between MTM and MTMSA-Trp observed in monkeys was lower than that in rodent models, we sought to understand whether this was related to small doses, i.e., microgram-level dose administration. Therefore, we conducted PK studies where we co-administered compounds at microgram-level doses to mice (69 µg/kg each of MTM and MTMSA-Trp) and rats (28 µg/kg each of MTM and MTMSA-Trp) (Studies 5 and 6; Table 1). These doses were estimated to be equivalent to a 12 µg/kg dose in monkeys using the estimation described in Section 2.3.4. Allometric scaling was used for its simplicity and ability to provide a reasonable approximation of interspecies dose extrapolation. Also, this approach is often recommended by regulatory guidance for initial dose estimation across different species [26]. Consistent with the previous PK studies in rodents and monkeys, MTM had a higher volume of distribution and a higher clearance than MTMSA-Trp (Table 4 and Figure 3). The systemic clearance of MTM was approximately 6.4-fold higher than that of MTMSA-Trp in mice and approximately 4.8-fold higher than that of MTMSA-Trp in the rats (Table 4). The rodent microdose PK studies showed that similarly to the monkey PK, the difference in clearance between MTM and MTMSA-Trp at microdoses is lower than at milligram-level doses (4–6-fold vs. ~20-fold) in mice and rats.

Comparing the MTM systemic clearance between microgram- and milligram-level doses, it decreased by approximately 1.4-fold and 4.7-fold in mice and rats, respectively (Table 3 and Table 4). In contrast, the MTMSA-Trp systemic clearance was approximately 0.4-fold lower in mice but remained similar in rats (Table 3 and Table 4). The faster systemic clearance of MTM at higher doses could be attributed to the non-linear behavior of plasma protein binding, in which the free fraction increases with an increasing drug concentration [27]. Due to sensitivity limitations, we did not measure the unbound fraction at concentrations lower than 1 µM and therefore did not experimentally verify it. It is plausible, however, that the unbound MTM concentration was much lower at microdose levels, sequestering the drug and resulting in lower systemic clearance. This may not have been the case for MTMSA-Trp, which appeared to be highly protein-bound across species at milligram-level doses. However, investigating plasma protein binding in the concentration range used in microdose PK studies could provide a better understanding of this phenomenon and may be a subject of future investigation if a more sensitive methodology becomes available. An alternative explanation could be related to drug–drug interaction since these studies were conducted in cassette mode. However, this is highly unlikely given the dose levels administered.

### 3.3. Dose Proportionality

As noted earlier, MTM analogs are likely to be OATP substrates [5]. Therefore, it is plausible that saturable hepatic uptake may lead to non-linear pharmacokinetics due to differences in liver partition and clearance. Thus, we tested whether MTMSA-Trp exhibits linear pharmacokinetics, or in other words, constant clearance and proportional exposure. The assessment of dose proportionality is critical for a better understanding of exposure–response relationships in efficacy studies. Since athymic nude mice have typically been used for anti-tumor efficacy studies for this class of compound, we chose this strain [5]. The MTMSA-Trp dose proportionality was evaluated in female athymic nude mice following the administration of 0.3, 1, 3, 5 and 10 mg/kg IV bolus doses (Studies 7, 8, and 9; Table 1). The estimated systemic clearance (Table 5) was similar at 1, 3, 5, and 10 mg/kg, with an average of approximately 41 mL/h/kg, and the clearance at 0.3 mg/kg was approximately 1.7-fold higher (69 mL/h/kg). This finding is consistent with the higher clearance observed at microdose levels, suggesting that MTMSA-Trp may exhibit non-linear PKs at lower doses. Notably, the maximum tolerated doses of MTMSA-Trp across different multi-day dosing schedules are in the range of 1–3 mg/kg [10], which is within the linear kinetics range of 1–10 mg/kg observed here.

## 4. Conclusions

This study investigated the interspecies differences in the pharmacokinetics of MTM and its analog, MTMSA-Trp. The results demonstrated that MTMSA-Trp exhibits higher plasma protein binding, leading to more favorable pharmacokinetics and increased plasma exposure compared to MTM in rodent models. The protein binding differences may have implications for their respective clearance mechanisms in the liver, which requires further investigation. Prolonged plasma exposure may allow for increased tumor biodistribution through convective transfer. However, increased protein binding may limit the free concentration of MTMSA-Trp in tumors, thereby compromising its efficacy, which will also necessitate prospective evaluation. Separately, MTM has rapid distribution and high clearance in humans, which was qualitatively recapitulated in rats. This warrants further investigation in the context of toxicity modeling to establish exposure–toxicity relationships among analogs. Collectively, these data may offer a basis for more advanced physiologically based pharmacokinetic modeling to predict the human pharmacokinetics of MTMSA-Trp.

## Figures and Tables

**Table 1:** Summary of pharmacokinetic studies.

Study	Species	Strain	Gender	Compound	Dose	No. of Animals	Time Points	Sampling Procedure
1	Mouse	Athymic nude	Male	MTM	2 mg/kg	15	5 min, 30 min, 1.5 h, 3 h, 6 h	Destructive
				MTMSA-Trp	2 mg/kg	15		
2	Rat	Sprague Dawley	Male	MTM	0.5 mg/kg	6	5 min, 20 min, 1 h, 3 h, 6 h, 9 h	Serial
				MTMSA-Trp	0.5 mg/kg	6		
3	Monkey	Cynomolgus	Male	MTM	6.94 µg/kg	1	5 min, 15 min, 30 min, 1 h, 2 h, 3 h, 4 h, 6 h, 8 h	Serial
				MTMSA-Trp	1.34 µg/kg	1	5 min, 15 min, 30 min, 1 h, 2 h, 3 h, 4 h, 6 h, 8 h	
4	Monkey	Cynomolgus	Male	MTM	13.18 µg/kg	1	5 min, 15 min, 30 min, 1 h, 2 h, 3 h, 4 h, 6 h, 8 h	Serial
				MTMSA-Trp	4.19 µg/kg	1	5 min, 15 min, 30 min, 1 h, 2 h, 3 h, 4 h, 6 h, 8 h	
5	Mouse	Athymic nude	Male	MTM and MTMSA-Trp ^1^	69 µg/kg ^2^	9	5 min, 15 min, 30 min, 1 h, 2 h, 3 h, 4 h, 5 h, 6 h	Serial
6	Rat	Sprague Dawley	Male	MTM and MTMSA-Trp ^1^	28 µg/kg ^2^	9		Serial
7	Mouse	Athymic nude	Female	MTMSA-Trp	1 mg/kg	12	5 min, 30 min, 2 h, 5 h	Destructive
					3 mg/kg	12		
					10 mg/kg	12		
8	Mouse	Athymic nude	Female	MTMSA-Trp	0.3 mg/kg	9	10 min, 20 min, 30 min, 45 min, 1 h, 1.5 h, 2 h, 2.5 h, 3 h	Serial
					1 mg/kg	9	5 min, 15 min, 30 min, 1 h, 2 h, 3 h, 4 h, 6 h, 8 h	
					3 mg/kg	9	5 min, 15 min, 30 min, 1 h, 2 h, 4 h, 6 h, 9 h, 12 h	
9	Mouse	Athymic nude	Female	MTMSA-Trp	5 mg/kg	10	10 min, 30 min, 1 h, 3 h, 6 h	Destructive

^1^ As cassette, ^2^ for each compound.

**Table 2:** Plasma protein binding of MTM and MTMSA-Trp in different species.

Plasma	Compound	Fraction Unbound (%)		
		1 µM	3 µM	10 µM
Mouse ^1^	MTM	4.61 ± 0.63	1.95 ± 0.14	1.08 ± 0.35
	MTMSA-Trp	<1	<1	<1
Rat ^2^	MTM	17.99 ± 1.57	15.15 ± 2.85	16.82 ± 1.21
	MTMSA-Trp	2.41 ± 1.29	3.85 ± 0.08	3.88 ± 0.54
Monkey ^3^	MTM	10.07 ± 1.34	12.75 ± 0.38	12.77 ± 1.19
	MTMSA-Trp	1.03 ± 0.27	1.02 ± 0.09	1.14 ± 0.15
Human	MTM	19.34 ± 1.57	18.28 ± 1.19	17.17 ± 0.70
	MTMSA-Trp	<1	<1	<1

^1^ Athymic nude/nude, ^2^ Sprague Dawley, ^3^ Cynomolgus macaque.

**Table 3:** Pharmacokinetic parameters of MTM and MTMSA-Trp in mice, rats, and monkeys.

Species	Gender	Compound	Dose	AUC_0–inf_ (ng/mL·h)	Extrapolated AUC_0–inf_ (%)	CL (mL/h/kg)	Terminal t_1/2_ (h)	V_d_ (mL/kg)
Mouse	Male	MTM	2 mg/kg	1905	6.74	1050	2.72	4125
		MTMSA-Trp	2 mg/kg	38,564	8.07	52	1.85	138
Rat	Male	MTM	0.5 mg/kg	175	53.52	2855	12.16	50,111
		MTMSA-Trp	0.5 mg/kg	3486	1.43	143	1.56	324
Monkey	Male	MTM	6.94 µg/kg	92	15.32	75	3.27	354
		MTMSA-Trp	1.34 µg/kg	139	5.77	10	0.37	5
		MTM	13.18 µg/kg	254	17.88	52	3.49	262
		MTMSA-Trp	4.19 µg/kg	181	4.99	23	0.78	26

**Table 4:** Pharmacokinetic parameters of MTM and MTMSA-Trp in male mice and rats at doses allometrically equivalent to 12 µg/kg in monkeys.

Species	Gender	Compound	Dose	AUC_0–inf_ (ng/mL·h)	Extrapolated AUC_0–inf_ (%)	CL (mL/h/kg)	Terminal t_1/2_ (h)	Vd (mL/kg)
Mouse	Male	MTM	69 µg/kg	89	1.3	777	0.7	821
		MTMSA-Trp	69 µg/kg	569	3.2	121	1.8	318
Rat	Male	MTM	28 µg/kg	46	3.1	608	0.8	713
		MTMSA-Trp	28 µg/kg	223	8.4	126	2.4	428

**Table 5:** Pharmacokinetic parameters of MTMSA-Trp in athymic nude mice dosed with ascending doses.

Dose	AUC_0–inf_	Extrapolated AUC_0–inf_	CL	t_1/2_	V_d_
	ng/mL·h	%	mL/h/kg	h	mL/kg
0.3 mg/kg	4373	0.4	69	0.5	45
1 mg/kg	24,419	0.2	41	1.3	75
3 mg/kg	91,778	0.2	33	2.6	123
5 mg/kg	13,7176	6.5	36	1.5	80
10 mg/kg	216,281	5.6	46	1.2	81

## Data Availability

Inquiries regarding the datasets should be directed to the corresponding author.

## Abbreviations

NCA	Noncompartmental analysis
C_last_	Last measurable concentration
AUC_last_	Area under the curve from time zero to last measurable concentration-time point
AUC_0–inf_	Area under the curve from time zero extrapolated to infinity
CL	Systemic clearance
V_d_	Volume of distribution
MRT_inf_	Mean residence time from time zero to infinity
PK	Pharmacokinetics
